# How clinicians integrate humanism in their clinical workplace—‘Just trying to put myself in their human being shoes’

**DOI:** 10.1007/s40037-018-0455-4

**Published:** 2018-10-08

**Authors:** Amanda Lee Roze des Ordons, Janet Margaret de Groot, Tom Rosenal, Nazia Viceer, Lara Nixon

**Affiliations:** 1 Departments of Critical Care Medicine, Anesthesiology, and Oncology (Div Palliative Medicine), Cumming School of Medicine, University of Calgary, Calgary, Alberta Canada; 2 Department of Psychiatry, Cumming School of Medicine, University of Calgary, Calgary, Alberta Canada; 3 Cumming School of Medicine, University of Calgary, Calgary, Canada; 4 School of Health Information Sciences, University of Victoria, Victoria, Canada; 5 Werklund School of Education and Department of Psychiatry, University of Calgary, Calgary, Alberta Canada; 6 Department of Family Medicine, Cumming School of Medicine, University of Calgary, Calgary, Alberta Canada

**Keywords:** Humanism, Clinical workplace, Attitudes, Behaviours

## Abstract

**Introduction:**

Humanism has been identified as an important contributor to patient care and physician wellness; however, what humanism means in the context of medicine has been limited by opinion and a focus on personal characteristics. Our aim was to describe attitudes and behaviours that enable clinicians to integrate humanism within the clinical setting.

**Methods:**

We conducted semi-structured individual interviews with ten clinical faculty to explore how they enact and experience humanism in patient care and clinical teaching. Interpretive description was used to analyze the data qualitatively.

**Results:**

Humanism in medicine was described through five themes representing core attitudes and behaviours: whole person care, valuing, perspective-taking, recognizing universality, and relational focus. Whole person care involved recognizing the multiple dimensions of personhood and sensitivity to others’ needs; valuing involved respecting and appreciating others; perspective-taking consisted of considering others’ perspectives, suspending judgment, and listening; recognizing universality involved acknowledging the shared human condition, finding common ground, transcending roles, and humility; and relational focus was described through multiple relationships between patients, families, clinicians and learners, becoming part of another’s story, reciprocal influence, and accompaniment.

**Conclusions:**

Whereas previous descriptions of humanism have focused on clinicians’ personal qualities, our research describes a number of attitudinal and behavioural foundations of humanistic care and teaching, grounded in the experiences of clinical faculty. In drawing attention to the holistic and relational elements of humanism, our work highlights how these foundational elements can be more explicitly integrated into patient care, workplace culture, and clinical education.

## What this paper adds

Humanism has been identified as a core tenet of patient care and physician wellness. Much of the literature has been centred around clinicians’ personal qualities. Personal qualities are less amenable to assessment, feedback and coaching, limiting our ability to explicitly integrate humanism into the formal curriculum. We identified attitudinal and behavioural aspects of humanism in the clinical setting, represented through whole person care, valuing, perspective-taking, recognizing universality, and relational focus. Attention to these domains may help clinicians more intentionally integrate humanism into their interactions with patients, and guide their approach to teaching and assessing humanism in working with learners.

## Introduction

Stories of patient dissatisfaction with medical care and reports of impaired wellness amongst physicians have sensitized the medical profession to gaps in our current approaches to patient care and medical education. The decline in attention to the experience and impact of illness on patients that has accompanied advances in the scientific and biomedical aspects of medicine was lamented many decades ago [1, 2], and remains an issue today [3–5]. For example, patients and their families express dissatisfaction with communication and emotional support in the medical care they receive [4, 6]. Within medical education, medical students and residents have noticed a lack of compassion in some of their preceptors’ interactions with patients [7, 8]; others report incidents of harassment and discrimination from preceptors, leading to psychological distress and impact on career choice [9]. Both trainees and physicians in practice experience high rates of burnout, characterized by emotional exhaustion, depersonalization, and a reduced sense of accomplishment, with personal and professional consequences [10, 11].

In response to these shortcomings in patient care and physician wellness, greater attention has been drawn to the relational aspects of medicine. Humanism describes the attitudes and behaviours that demonstrate interest in and respect for patients’ psychological, social and spiritual concerns and values [12]. Previous opinion papers and research have been valuable in conveying the importance of humanism and the qualities of humanistic clinicians [13, 14], yet how clinicians translate these principles into their daily clinical work has not been well described [15]. One study analyzed the written narratives of physicians who were considered to be ‘highly humanistic’ and identified that they responded to challenges with intuitive and/or deliberate behaviours that strengthened relationships and benefited others, such as recognizing distress in patients and learners, and responding by offering comfort or sharing their own experiences, respectively. Furthermore, their actions were aligned with and reaffirmed deeply held beliefs and values, for example, compassion and integrity [16]. Another study focused on teaching described how physicians role model humanism through non-verbal communication, respect, personal connection, responding to emotion, and self-awareness [17]. A better understanding of the attitudes and behaviours that enable clinicians to integrate humanism within the complexities of the clinical setting would inform approaches to addressing gaps in humanistic care. We set out to explore how clinicians recognized for their humanistic qualities integrate humanism into patient care and medical education within their routine clinical teaching practice environments.

## Methods

We adopted qualitative methods to explore clinicians’ perceptions of what humanism means in clinical medicine. Physicians attending a longitudinal course on teaching humanism and professionalism at the University of Calgary between April and June 2015 were invited to participate in the study. The physicians attending the course had been identified by their department heads as being recognized for their humanistic qualities. The course was adapted from a previously described faculty development program at Emory University [18, 19]. One member of the research team (NV) conducted semi-structured interviews of 20 to 40 minutes by telephone or in person at the start of the course, using a guide the research team had prepared based on the study objectives and related literature (Appendix 1). Participants were asked to describe what humanism means to them, its importance in the practice of medicine, and a time when they witnessed or provided humanistic care. Interviews were audio recorded, professionally transcribed verbatim, and anonymized.

The research team consisted of an interprofessional group of medical educators experienced in qualitative research and with collective backgrounds in family medicine, palliative care, psychiatry, anaesthesiology, and critical care medicine. Two members were also facilitators for the humanism and professionalism course (LN, TR), and ALR and JMdG attended the course as participants. All research team members except NV had clinical or medical education working relationships with the course participants.

With a social constructionist view that social and interpersonal influences shape experiences and interpretations of the world [20], we analyzed the transcripts using interpretive description [21], and with a focus on attitudes and behaviours that conveyed humanism. Interpretive description emphasizes grounding research within the social context of clinical settings such that findings can inform practice and education; data interpretation builds on prior knowledge and experience of the clinical setting where the research is conducted to develop a broader understanding of a phenomenon [21]. Two members of the research team with different clinical backgrounds (ALR, LN) independently read through the transcripts to become familiar with the data and inductively coded the transcripts line by line to generate a codebook of humanism-related attitudes and behaviours. Each code was accompanied by a definition and supporting quotes. The coding team met after coding every two transcripts to discuss and refine codes; discrepancies were resolved through discussion. Related codes were grouped into themes and subthemes, and organized into a framework. The themes and subthemes were then applied to the entire data set. Data analysis was also informed by notes taken during debriefing amongst research team members following each session of the course and memos written throughout the coding process. Ethics approval for the study was obtained from the University of Calgary Conjoint Health Research Ethics Board.

## Results

Ten of the 12 course attendees participated in the study. Six participants were women and four were men, with representation from internal medicine and its subspecialties, surgical subspecialties, psychiatry, and paediatrics. Participants’ clinical and teaching experience ranged from 2 to over 25 years.

We identified five themes that describe attitudes and behaviours through which clinicians integrate humanism into their clinical practice: whole person care, valuing, perspective-taking, recognizing universality, and relational focus. There were multiple subthemes that we describe below, accompanied by illustrative quotes. In the text below, subthemes are indicated in italics.

### Whole person care

Participants described how they acknowledge the physical, cognitive, psychological, emotional, social and spiritual *dimensions* of a person, as compared with the narrow focus on the physical elements of disease that often dominates medical practice.

*Looking at people as their entire person, looking after their social, emotional, physical, psychology, the well-being of the entire person … not just necessarily the physical*. (Transcript 1)

*I think it’s very important that we don’t regard our patients as ‘cases,’ you know? That we see the person and all their human qualities and their interactions and their families and who they are as well as the thing that we’re trying to fix or treat or whatnot, rather than seeing, you know, understanding them in isolation as just, okay, this person has this problem that I need to fix*. (Transcript 7)

Humanism involved *sensitivity* to the other person’s needs in the context of their illness and their life story.

*I think [humanism] is a sense of understanding the human condition and being sensitive to the needs of the person and the individual as a whole in the context of their life and what they’re going through*. (Transcript 2)

*On a day-to-day basis when I see patients … take into account the sphere of their illness and the context in which they find themselves … I make certain that they are able to afford their medications, they are able to have home care if need be so that the optimum care can be provided given the circumstances they find themselves in at that point in their lives*. (Transcript 7)

### Valuing

Clinicians further conveyed humanism through a profound *respect* for a person’s intrinsic value. This concept was particularly apparent in stories of patients who were marginalized by their social status or diagnosis.

*He was a street person, he was down and out, he had a fairly significant medical problem with possible vertebral osteomyelitis, he had been bounced around from clinic to clinic and the emergency room, but I took the time … I have always felt that you have to treat each person equally regardless of their social status in life, you shouldn’t treat anybody who is the premier of the province any differently from the guy who comes in off the street. You’re there as a physician regardless of where they come from or what the nature of their illness is.* (Transcript 9)

The capacity to *appreciate* others for their intrinsic qualities further characterized the concept of valuing in humanistic care, or believing in *‘the inherent value of human beings, the goodness of human beings’ (Transcript 7), *as illustrated below*.*

*The patient had addiction issues and HIV and a lot of the efforts at the end of that person’s life were directed at trying to respect what he valued and what he would have wanted, although he could not participate … Contacting people that he interacted with, a friend, a sister, and making sure that we were respecting him … Although, you know, he had a lot of … he looked intoxicated, he’s a drug abuser, the disease was due to the drugs, so if you take it just from the outside, some people maybe would have said, ‘Well, you know, it’s kinda your fault,’ and when he was belligerent, you’d say, ‘Well, if you’re intoxicated, don’t come bug me,’ but it was just completely the opposite that took place. He was respected across that experience*. (Transcript 6)

### Perspective-taking

*Considering others’ perspectives* was another foundational element of clinicians’ humanism.

*When I’m working with somebody, communicating with them or trying to help them, it’s me putting myself in their shoes and imagining … just trying to put myself in their human being shoes and, and maybe speak with them or treat them and have compassion towards them like I would want to be treated if I were in their shoes*. (Transcript 4)

*One thing that I think is really important is having time to spend with people who need more time and understanding that they have a perspective which is not always the same as the healthcare provider’s perspective and respecting that and trying to work with them.* (Transcript 7)

Perspective-taking also involved a willingness to *suspend judgment*. One participant described how they apply this principle in working with patients who have ‘a different world view’.

*I have found that many of my colleagues are quite dismissive of patients who want to follow complementary or alternate medicine, rather than the scientifically based or biomedical model that physicians are trained in and that we follow, and over the years …I’ve become very interested in this area of complementary treatment and demonstrate to patients I’m open to what they’re interested in and what they might want.* (Transcript 7)

*Listening* to patients’ and their families’ views, their hopes and their fears, and helping them to reconstruct their life story were ways through which clinicians came to understand what patients and their families were experiencing.

*An indigenous patient who was dying in the ICU and, like, fifty people showed up to the family meeting and just hearing everyone’s perspective was very interesting. I think my preceptor had been in that situation before and he knew how much that meant to the community and this person was a big important part of their community.* (Transcript 5)

*We were talking about withdrawal of life sustaining therapies in a patient with a traumatic brain injury and the attending that I was working with at the time, rather than talking about the diagnosis and all of the implications, he started out with just addressing how horrible and awful the situation was and how gut-wrenching it was for the family to watch someone that they loved dying … and he spent time with the family and sat with them and heard their concerns, sat with them through that grieving process.* (Transcript 10)

### Recognizing universality

Clinicians were able to relate to their patients’ experiences through *acknowledging the shared elements* of the human condition, *finding common ground*, and *transcending the separation* imposed by professional boundaries.

*To me I think it’s … a sense of understanding the human condition.* (Transcript 10)

*How human beings all have common needs and understanding …the importance of acknowledging that human dimension of everyone and everyone with a common need*. (Transcript 7)

*The only way that you can understand each other and move forward is if you try to understand them as a human being and where they’re coming from, and you know what, what the experience they’re going through is like for them. And without that, you just become kind of one-sided and a little bit selfish in terms of the goal at hand and aren’t really accomplishing what truly needs to be accomplished.* (Transcript 4)

There was a sense of *humility* in honouring this shared humanity and the associated fallibility and vulnerability that both patients and clinicians might experience at times. One participant described observing a colleague’s interaction with a family after a procedural complication.

*And thankfully it didn’t cause the patient significant harm and it was very easily rectified, but it was extremely upsetting for the family who were very upset and nervous about the whole procedure needing to even happen. And so I witnessed that person coming to the family in a very humble way, providing them with information but being very honest and compassionate the way that he spoke with them, and apologetic. But also just laying out for them the human side of himself and that he had deeply reflected about what happened, and trying to regain their trust.* (Transcript 4)

### Relational focus

The clinicians’ narratives described *multiple relationships* between clinicians, patients and their families, colleagues, learners, and within themselves.

*Coming in to work every day and trying to remember that not just my patients, but my colleagues as well as my trainees, we’re all human*. (Transcript 8)

*The human aspect of everything we do, the influence of being human on our activities and vice versa, the impact of our activities or actions on us as human beings.* (Transcript 3)

Humanism also involved *becoming part of another person’s story*.

*I took the time and effort and he seemed genuinely appreciative … he stopped me at the end and said, ‘You’re the first person who’s ever taken the time and interest to be able to find out exactly what’s wrong with me without bouncing me around given my circumstances in life’ and so I was quite touched that he actually came out and said that.* (Transcript 9)

Clinicians had a self-awareness that in entering into another person’s world, they were themselves *reciprocally influenced* and at times transformed.

*Understanding that you yourself are a human being and kind of embracing the emotion and the feelings that you’re feeling while doing that.* (Transcript 4)

*There needs to be some additional emphasis in medical training on the development of skill sets for coping with difficult situations … to help practitioners deal with their own emotional responses to emotionally challenging and difficult situations*. (Transcript 10)

Participants reflected that meaningful connection helped clinicians *accompany* patients and their families genuinely and support them in a caring way through times of difficulty.

*I find the social worker to be amazing at really kind of understanding where patients and their families are coming from, what are their, you know, fears, what are their hopes. She’s really good at integrating what we’re saying to people in terms of the medicine and integrating it into their entire kind of life story*. (Transcript 1)

*Back in my training I used to accompany my professor doing afternoon/evening rounds with patients … I was very impressed with the care and compassion that he provided, and I guess I have tried to, where possible, model or imitate that approach. The impact on the patient I saw at that time, and still continue to see, was a reduction of the stress and the agitation, and a sharing of the burden of suffering.* (Transcript 2)

At other times, support involved interactions between clinicians and learners, as described below.

*The poor resident came out and was in tears … what was particularly nice to do was to sit down with the resident afterwards and we had a really thorough discussion about what just happened.* (Transcript 3)

## Discussion

We have described how clinicians integrate humanism into clinical medicine through attention to whole person care, valuing, perspective-taking, recognizing universality, and a relational focus. While previous authors have largely defined humanism in medicine on the basis of their own personal experiences and with emphasis on intrinsic characteristics [13, 15], our study reveals explicit and actionable attitudes and behaviours that enable clinicians to integrate humanism within clinical medicine. Our findings have implications for patient care, workplace culture, and competency-based medical education.

The notion of humanism as being focused on the patient as a whole reminds us that medicine encompasses more than the diagnosis and treatment of physical ailments. Indeed, the cognitive, psychological, emotional, social and spiritual dimensions of clinical interactions impact both patients and clinicians. While bio-psycho-social-spiritual models of patient care have been described [22, 23], implementing such models in clinical practice remains a challenge. Recognized challenges include time constraints, work-related and personal stress, organizational culture, and episodic burnout [24]. Burnout impairs the quality of patient care through clinician detachment from their work [25, 26]. The humanistic behaviours and attitudes reported by clinicians in our study helped them feel connected to patients and their families, and to learners. This research adds to the discourse around the notion that humanism may reduce burnout and enhance patient care through helping clinicians find meaning in their work and fostering resilience [24, 27].

The clinical workplace encompasses complex interactions between multiple individuals and teams. Yet previous accounts of humanism in clinical medicine have been clinician-focused [7, 15, 17] or teacher-focused [28, 29], describing specific clinician characteristics as humanistic, and considering clinician behaviours directed towards the patient or learner; there is a sense of separateness and unidirectionality. Our work draws attention to the relational foundation of humanism, where others’ perspectives and contributions are valued, and where clinicians, patients and learners are reciprocally impacted by their interactions and acknowledge their shared humanity. These elements align with the concept of relationship-centred care [30]. Relationship-centred care has been described as integrating four elements of relationships in healthcare: honouring individual personhood, affect and emotion, reciprocal influence, and the moral value of these relationships [30]. Our work serves as a reminder that humanism has relevance beyond the patient-clinician relationship and is equally applicable to relationships with colleagues, learners, and with the self. Applying the attitudes and behaviours described in our study to this broader set of relationships has implications for workplace culture, where humanistic interactions with colleagues and learners have the potential to improve teamwork and collegiality. Our findings illustrate how self-awareness is also a core element of humanism. The capacity for self-awareness, where healthcare providers are able to recognize how they themselves are impacted by their interactions with patients, has been well-described in the psychiatry literature through concepts of transference and countertransference [31].

Our findings have additional implications for competency-based medical education. Considering the complexity of humanism in the healthcare setting, the assessment of humanism will require a multidimensional approach. Observing behaviours alone does not consider the thought processes and internalized attitudes nor reveal the tensions that a clinician may be grappling with and navigating [32, 33]. In a meta-narrative systematic review of measures of quality in therapeutic relationships, Greenhalgh and Heath emphasized that no single metric can be relied upon to evaluate the complex and dynamic nature of humanistic care [34]. With assessments in competency-based medical education being behaviourally-based [35], our description of humanism reminds educators of the need to supplement observations with dialogue around attitudes, and the importance of considering the reciprocal impact of behaviours in addition to the behaviours themselves.

Our study has limitations. We focused on the perspectives of clinical faculty with a specific interest in humanism. Obtaining perspectives from other healthcare providers, learners, and patients could provide a broader and more in-depth understanding of humanism in the clinical setting. The small number of participants within a single Canadian institution limits the transferability of our findings. We recognize that how humanism is conceptualized will vary by culture and context, and we do not suggest that this paper provides a universal set of principles. What we do hope is that it offers a foundation upon which to ground future conversations about the complexity of how humanism is enacted and experienced, how it can be learned and assessed within contemporary healthcare settings, and the tensions that arise therein.

## Conclusions

Grounded in clinicians’ experiences of integrating humanism within their workplace settings, our study describes a set of relationship-centred attitudes and behaviours that foster and reify humanism in interactions with patients, colleagues, learners and the self. These findings encourage a critical examination and creative imagining of how embedding humanism within clinical medicine has the potential to transform patient care, workplace culture, and clinical education.

## Appendix I

### Focus group and interview guide

**I. General questions**

1. What drew you to medicine?
2. What aspects of your medical/surgical practice energize you now?
  - Probes: a. What is it about this aspect that energizes you?
3. What aspects of your current medical/surgical practice inspire you?
  - Probes:a. What is it about this aspect that inspires you?

**II. Humanism**

1. What does humanism mean to you?
2. In what way(s) is humanism important in the practice of medicine?
  - Probes: a. Can you tell me more about that? b. How are humanistic principles currently integrated into your work environment?
3. Please tell us about a time that stands out for you when you provided or witnessed humanistic care for a patient.
  - Probes: a. What was going on at the time? Where were you? Who else was there? b. How did you feel? How did the patient feel? c. What was the impact on the patient and on you? d. What did you learn from this experience?
4. How could humanistic principles be routinely integrated into your workplace setting?
  - What would you like to see more of?
  - What would need to change to make this happen?
  - How could you help bring about these changes? What support would you need?
  - What might be the result?
